# Randomized controlled trial comparing embryonic quality in rFSH *versus* hMG in the IVF protocol with GnRH Antagonist

**DOI:** 10.5935/1518-0557.20200064

**Published:** 2021

**Authors:** Rita de Cassia Borges Chapon, Vanessa Krebs Genro, Carlos Augusto Bastos de Souza, João Sabino Cunha-Filho

**Affiliations:** 1 Universidade Federal do Rio Grande do Sul, Programa de Pós-Graduação em Medicina: Ginecologia e Obstetrícia. Obstetrics and Gynecology Department, Porto Alegre, RS, Brazil; 2 Hospital de Clínicas de Porto Alegre, Universidade Federal do Rio Grande do Sul, Obstetrics and Gynecology Department, Porto Alegre, RS, Brazil; 3 Professor of the Department of Obstetrics and Gynecology, Universidade Federal do Rio Grande do Sul Obstetrics and Gynecology Department, Porto Alegre, RS, Brazil.

**Keywords:** *In vitro* fertilization, rFSH, hMG, GnRH antagonist, embryonic quality

## Abstract

**Objective:**

The aim of the present study is to investigate embryo quality (score) after controlled ovarian stimulation for IVF using rFSH or hMG with the GnRH antagonist protocol.

**Methods:**

Open, randomized, single center study. The patients were randomized to receive rFSH or hMG according to randomized cards inside a black envelope with the name of the respective treatment following a computer generated list (85 patients were allocated to rFSH group and 83 patients to hMG group). Inclusion criteria were patients with IVF indication and normal ovarian reserve. Embryo evaluation was performed on day three, after fertilization based on the Graduated Embryo Score (GES).

**Results:**

There were no relevant differences in demographic characteristics. There was no difference in pregnancy rates with 27 (31%) and 25 (30.1%) pregnancies for rFSH and hMG, respectively (*p*=0.87). The total embryo score was the same for both groups, but the best embryo score was significant higher for the rFSH group (77.33±34.0 x 65.07±33.2 *p*=0.03). The total number of embryos was statistical different, also in favor of the rFSH group (4.17±3.1 x 3.26±2.4 *p*=0.04).

**Conclusion:**

The total embryo score was the same for both groups, but the best embryo score was significantly higher for the rFSH group. Moreover, rFSH was associated with an increased number of embryos.

## INTRODUCTION

We still do not completely understand the specific action of each gonadotropin in the folliculogenesis. The difficulty to understand their differences is even greater when we consider the heterogeneity of studies regarding the design, group of patients and type of protocol used to prevent the ovulation (GnRH agonist or GnRH antagonist). Because the GnRH agonist was the first analog to be used in IVF cycles, there are more studies with this protocol. In recent years the GnRH antagonist has been even more utilized in IVF protocols, but there is a limited number of studies comparing recombinant follicle stimulating hormone (rFSH) and human menopause gonadotropin (hMG) when this protocol is used (Karlström *et al.*, 2018; Olivennes *et al.*, 2002).

Some studies have demonstrated differences in clinical IVF outcomes, such as number of oocytes retrieved and number or quality of embryos when comparing rFSH and hMG to ovarian stimulation. A correlation between the number of oocytes retrieved (Bosch *et al.*, 2008) and a higher number of embryos (Bjercke *et al.*, 2010) when using the rFSH have already been detected in some studies, but without detectable difference in pregnancy rates.

Other studies showed opposite results and better outcomes with hMG. In 2008 a meta-analysis compared hMG and rFSH in the long GnRH agonist protocol. They analyzed seven randomized trials, including 2259 IVF cycles, and found a significant increase in life birth rates with hMG when compared to rFSH, with a relative risk of 1.18 (Coomarasamy *et al.*, 2008). None of these seven trials individually showed a statistically significant benefit towards hMG, although five of them showed a trend in favor of hMG.

Besides the lower number and heterogeneity of studies analyzing the GnRH antagonist protocol, we see that most of the studies are not well designed. Most of these studies used pregnancy rates as the main outcome, even when they do not enroll a significant number of patients to find statistical differences in the results. Thus resulting in an underpowered study, since the number of patients needed to find statistical differences in these outcomes would be 2,400, and we did not find this in our literature review. Hence, the few studies that enrolled a higher number of patients declare themselves sponsored by the pharmaceutical industry (Andersen *et al.*, 2006).

Since embryonic quality is considered to be in direct correlation with pregnancy rates, we chose to set it as our main outcome. Few studies have compared this relevant predictor of IVF success. In addition, the number of patients needed to achieve a high statistical power is lower than the number needed to find differences when comparing pregnancy rates, which makes it suitable for our research center. Some authors demonstrated that blastulation could be linked to hMG administration, although the mechanism was not clear (Ziebe *et al.*, 2007).

Considering the controversial results until now and the lack of knowledge in this important field of reproductive techniques, we aimed to better understand the differences in ovarian stimulation comparing embryonic quality with rFSH and hMG in IVF cycles with GnRH antagonist protocol.

## MATERIALS AND METHODS

### Design

This was a randomized, open-label, single-center controlled study to compare hMG (Menopur^®^, Ferring Pharmaceuticals, Denmark) and rFSH (Puregon^®^, Organon Ltd., Ireland) in patients undergoing ovarian stimulation for IVF using the GnRH antagonist protocol. Our patients were randomized 1:1 to receive rFSH or hMG according to randomized cards inside a black envelope with the name of the respective treatment following a computer-generated list. The present study was included in the Clinical Trials protocol registration system - NCT02412904. We used the CONSORT statement for RCT.

### Patients and Sample Size Estimation

Infertile patients from a single center of reproductive medicine with indication for IVF were randomized to receive hMG or rFSH for ovarian stimulation. The patients were invited to participate if they met all the following criteria of normal ovarian reserve: normal FSH <10 mUI/ml; Anti Müllerian Hormone (AMH) between 1 and 3 ng/ml; AFC (Antral Follicle Count) >12; and regular menses (25-35 days).

Patients were excluded if they had endocrine pathologies, severe masculine factor (all patients were submitted to IVF without ICSI), previous pelvic surgery or ovarian cysts.

We estimated the sample size using a significance level of 0.05 and a power of 80% to detect a relevant difference in the embryonic quality between groups, based on a previous study (Bosch *et al.*, 2008).

The study started after the approval from the Ethical Committee. All patients were informed that the study would not interfere or present any risks for their treatment.

### Intervention and Protocol

All the patients had their ovarian reserve analyzed (AMH and AFC), and an ultrasound scan performed to rule out ovarian cysts and other pelvic abnormalities prior to treatment. After the complete evaluation, the patients were asked to schedule an ultrasound scan at the beginning of the menstrual cycle (first three days). At this time, we started ovarian stimulation with rFSH or hMG (according to previous randomization), using a dose between 150-300IU according to their AMH and AFC. This dose was maintained until day 6 of stimulation, when a second ultrasound scan was performed and the GnRH antagonist (0.25mg Ganirelix, Orgalutran^®^ Merck Sharp & Dohme, Australia) was initiated and continued until the end of the cycle. Seriated ultrasound scans were performed every other day and the hCG (human chorionic gonadotropin, 5,000 IU Choriomon^®^ IBSA Institut Biochimique S.A., Switzerland) was administered when at least three follicles reached 17 mm of mean diameter. We retrieved oocytes 36 hours afterwards.

We assessed the embryos on day three after fertilization, based on the Graduated Embryo Score (GES) (Fisch *et al.*, 2001). Three evaluations were performed at 16-18 hours, 25-27 hours and 64-67 hours post fertilization, respectively, by the same embryologist who was blinded for the intervention. The score was composed by the following criteria: nucleolar alignment along pronuclear axis, regular cleavage and degree of fragmentation at the first cell division, and cell number and morphology on day 3 after fertilization. The maximum score was 100. The total score was calculated by the sum of embryo scores. The embryos were transferred on day 3. The patients were advised to have a pregnancy test done 12 days after the embryo transfer.

All outcomes (dose of gonadotropins, number and size of follicles, number and score of embryos) were registered during cycles by a restricted and trained team of three doctors and two embryologists.

This study was not sponsored by the pharmaceutical industry.

### Objective

To compare embryonic quality and other clinical outcomes in IVF cycles with GnRH antagonist protocol using human menopause gonadotropin (hMG) or recombinant follicle stimulating hormone (rFSH).

Our primary outcome was embryonic quality, based on total embryo score and best embryo score. The embryo evaluation was performed based on the Graduated Embryo Score. Moreover, the secondary outcomes were total dose of gonadotropins, number and size of follicles at the end of the ovarian stimulation, number of retrieved metaphase II oocytes and clinical pregnancy rates.

### Statistical Analysis

We ran the statistical analysis using the SPSS 20^®^ software, applying the Student T-test for independent samples and the Levene test for equality of variances. Data are expressed as mean ± standard deviation (SD) for continuous variables, and as mean and 95% confidence interval (95%CI) for categorical values. We ran a multivariable analysis to investigate confounding factors.

## RESULTS

**Figure 1:**
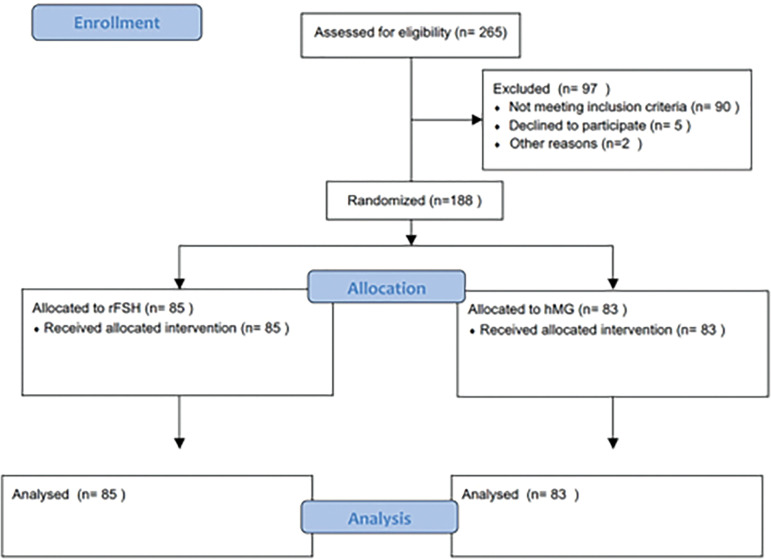
CONSORT Flow Diagram

A total of 265 patients were eligible (flowchart), 97 were excluded because they did not fulfill the inclusion criteria or they refused to participate in the study. One hundred and sixty eight patients were randomized, 85 patients were allocated to the rFSH group and 83 patients to the hMG group (Figure 1). There were no relevant differences in the demographic characteristics including mean age, body mass index (BMI) and ovarian reserve tests (Table 1).

**Table 1:** Baseline characteristics (values expressed by mean ± Standard Deviation, and number (%) for categorical variables)

Baseline Parameters	rFSH (n=85)	hMG (n=83)	*p*-value
Age (years)	34.4±2.5	34.9±2.3	0.22
AMH (ng/ml)	4.3±3.3	3.5±3.1	0.29
FSH (mIU/l)	7.1±2.3	6.4±2.0	0.10
AFC	12±1.7	14±1.5	0.13
BMI (m/Kg^2^)	24.4±1.6	23.7±1.8	0.86
Primary cause of Infertility			0.73
Male Factor	29 (34.1%)	27 (32.5%)	
Tubal Infertility	28 (32.9%)	27 (32.5%)	
Endometriosis	28 (32.9%)	24 (28.9%)	

The major causes of infertility were tubal factor (35.7%), masculine factor (33.3%) and endometriosis (30.9%), without differences in the distribution between the studied groups.

All the patients had at least one embryo transferred on day 3. The maximum number of embryos to be transferred was decided in accordance to patients’ desire and age. Patients under 30 years had only 1 embryo transferred, 31 to 35 years 1 to 2, over 36 years 1 to 3.

The total embryo score was the same for both groups but the best embryo score was significantly higher for the rFSH group (77.33±34.0 x 65.07±33.2, *p*=0.03). The total number of embryos was statistical different, also in favor of the rFSH group (4.17±3.1 x 3.26±2.4, *p*=0.04).

Table 2 shows the ovarian stimulation outcomes. Differences in pregnancy rates were not seen among these 27 (31%) and 25 (30.1%) pregnancies for the rFSH and the hMG respectively (*p*=0.87). Considering the other secondary outcomes, the total dose of administered gonadotropins, number of MII oocytes and size of follicles, no statistical difference was detected.

**Table 2:** Ovarian stimulation outcomes (values expressed by mean ± SD)

Outcome	rFSH (n=85)	hMG (n=83)	*p*-value
Total gonadotropin dose (IU)	3033±663.50	2976±628.4	0.58
Follicles 13-14 mm	0.99±0.99	1.01±1.0	0.92
Follicles 15-16 mm	3.45±3.0	3.62±3.3	0.73
Follicles ≥17mm	5.24±3.4	4.76±3.0	0.35
Number of metaphase II	6.12±3.1	5.35±2.5	0.08
Number of embryos	4.17±3.1	3.26±2.4	0.04
Total embryo score	214.01±162.4	170.43±176.6	0.13
Best embryo score	77.33±34.0	65.07±33.2	0.03
Clinical pregnancy/randomized patient	27/85 (31.7%)	25/83 (30.1%)	0.87

The logistic regression model also confirmed that the number of embryos and the best embryo score (day-3) were linked to rFSH administration during controlled ovarian stimulation.

## DISCUSSION

Many studies have compared rFSH and hMG in IVF cycles regarding their effectiveness in ovarian stimulation. Since hMG has a different composition, concerning the presence of LH, it has been speculated that this would affect the outcomes in follicular recruitment, follicular growth, number and quality of embryos and pregnancy rates (Bordewijk*et al.*, 2019; Zeleznik & Kubik, 1986; Ziebe*et al.*, 2007).

There are few studies comparing rFSH and hMG for IVF with the GnRH antagonist protocol. Our group found some statistical differences in the pattern of ovarian stimulation of these two gonadotropins. There was a higher number of embryos, and a higher score of embryo quality in the rFSH group, despite the fact that no difference was detected in the number of retrieved MII oocytes. This could reflect a role of the LH activity in the follicular phase that may cause some negative impact on oocyte quality. We know that the LH/hCG is involved in the process of oocyte atresia (apoptosis), so this mechanism could also interfere in oocyte quality and thereafter in embryo quality (Hirata *et al.*, 2015).

Our findings are in accordance to previous clinical randomized trials that compared rFSH and hMG in GnRH antagonist cycles (Bordewijk*et al.*, 2019; Bosch *et al.*, 2008). They observed a lower number of oocytes retrieved with a mean difference of 3.1 in favor of the hMG group, and a lower number of MII oocytes with a mean difference of 1.9. The lower number of retrieved oocytes in hMG group was also explained by the LH effect during follicular phase, and its involvement in the atresia process. They found no differences in pregnancy rates.

These findings also coincide with studies that included the GnRH agonist protocol, including the Merit study (Ziebe *et al.*, 2007) which showed a significantly higher number of oocytes retrieved in the rFSH group. Despite the lower number of oocytes in the hMG group, and differently from our findings, they detected the best embryonic quality in this group. The influence of the long agonist protocol, that causes a more intense pituitary suppression than the GnRH antagonist, could have impacted this controversial result. Despite these differences, pregnancy rates did not differ between rFSH or hMG, which was confirmed recently in a meta-analysis (Bordewijk *et al.*, 2019).

Our study found similar pregnancy rates for both groups, which is in accordance with the few studies that have included this specific IVF protocol (Bosch *et al.*, 2008; Devroey *et al.*, 2012). Although the efficacy of both drugs did not differ, we found some particularities in their profile of ovarian stimulation that need to be better understood and will be further discussed.

The variability of IVF protocols and patients’ profiles has complicated the studies in this field. Most of these studies included patients that used the GnRH agonist protocol, mainly the long protocol (Andersen *et al.*, 2006; Bjercke *et al.*, 2010; Olivennes *et al.*, 2002). The results are very controversial in terms of hormonal profile during ovarian stimulation with some differences in follicular recruitment and embryonic quality. Platteau *et al.* (2004) analyzed 727 IVF cycles with the GnRH agonist protocol and found more oocytes retrieved in the rFSH group, despite a significant more positive beta-hCG test in the hMG group of IVF patients. This result was not seen in the subgroup analysis of the patients submitted to ICSI. They speculate that the LH activity could have a beneficial effect on the pregnancy rates in women undergoing IVF, when the female factor is the main cause of infertility.

Our study was carried out in a single center with a restricted number of researchers, which has the advantage of avoiding potential confounding factors and some biases such as dose adjustment policy, ultrasound measures and embryo assessment criteria. We intended to minimize the bias of the hormonal profile of the patients considering the influence of the response to exogenous gonadotropins, so we included only women with regular menses, normal ovarian reserve tests and no endocrine diseases.

There is a lack of information whether or not the LH activity in ovarian stimulation preparations improves the outcomes in IVF. Is it beneficial for some specific population? A Cochrane systematic review in 2007 analyzed 14 clinical trials (eleven of them using the GnRH agonist); including 2,612 patients and they compared rFSH *versus* rFSH plus recombinant LH (rLH). There was no statistical difference in pregnancy rates, but three trials, that included only poor responders, showed significant increases in pregnancy rates, favoring the co-administration of rLH, and these results were confirmed more recently by others authors (Minareci & Ozcan, 2019; Mochtar *et al.*, 2007; Shahrokh Tehraninejad *et al.*, 2017).

In conclusion, we had results that statistically differed in the number of embryos and the best embryonic score in favor of the rFSH group. We suppose that gonadotropins might have some impact in oocyte and embryo quality, maybe because of some interference of the LH/hCG presence in hMG preparations. Further studies are needed to better explain these findings.
